# Preoperative Stress Testing before Non-Cardiac Surgery

**DOI:** 10.31083/j.rcm2404098

**Published:** 2023-03-23

**Authors:** Olga Dzhioeva, Marijana Tadic, Evgeny Belyavskiy

**Affiliations:** ^1^Department of Therapy and Preventive Medicine, Moscow State University of Medicine and Dentistry named after A.I. Evdokimov, 127473 Moscow, Russia; ^2^Klinik für Innere Medizin II, Universitätsklinikum Ulm, 89081 Ulm, Germany; ^3^Department of Cardiology, Angiology and Intensive Care Medicine, Deutsches Herzzentrum der Charité (DHZC), 13353 Berlin, Germany

**Keywords:** non-cardiac surgery, perioperative risk assessment, cardiovascular complications, stress-test, stress echo before non-cardiac surgery

## Abstract

The current guidelines from various medical societies provide a good summary of data regarding various preoperative exercise tests in patients prior to non-cardiac surgical interventions. However, there is no consensus among experts on the appropriateness of these methods for identifying risk groups for potential perioperative complications. A large volume of published studies describes the role of preoperative exercise stress testing impact in improving the prediction of potential cardiovascular (CV) risk in patients after non-cardiac surgery. Numerous stress tests are available in clinical practice, and the methods used and the best choice depends on the purpose of the study and the availability of equipment in the hospital. Traditionally, the value of exercise electrocardiography (ECG), or ECG stress test, has been based on the belief that it is beneficial for perioperative cardiac risk prediction. However, in the past two decades, the key role of this method has lost its importance due to the growing trend toward the use of imaging techniques. Moreover, in light of current trends, the six-minute walk test (6MWT) is a helpful tool in preoperative assessment and plays an important role in postoperative rehabilitation. Interestingly, the recent finding showed how 6MWT affects the risk of postoperative complications. Cardiopulmonary testing, as a dynamic clinical tool, determines the cardiorespiratory status of a patient. Various clinical indications for cardiopulmonary exercise testing include evaluation of therapy, stratification of risk factors, diagnosis of disease, and control of physical activity. Stress testing is one of the most practical ways of predicting perioperative risk and managing patients. This test is based on ischemia provoked by pharmacological agents or exercise. There is no established evidence of a significant advantage of pharmacological stress over exercise stress imaging in subjects who are capable enough to be physically active. All of these studies examined a stress test for induced myocardial ischemia. Currently, there are no data on the use of ischaemic stress tests, especially diastolic stress tests, in the assessment of perioperative risk before non-cardiac surgical interventions. We consider it promising and essential to continue research in this direction in patients with coronary heart disease and other categories of cardiac patients, in particular, comorbid and low-symptomatic individuals, before elective high-risk surgical interventions.

## 1. Introduction

Despite current recommendations for the stratification of cardiac complications of cardiac complications in non-cardiac surgery, preoperative risk assessment remains one of the most difficult clinical tasks and decisions. There is disagreement among physicians about perioperative risk assessment, especially in patients without a history of previously diagnosed cardiovascular (CV) events. Moreover, patients with verified or symptomatic cardiac pathology can lack complete preoperative examination if they have no significant hemodynamic disorders and clinical symptoms at rest. In late August 2022, the European Society of Cardiology (ESC) published its latest guidelines on cardiovascular assessment and management of patients undergoing non-cardiac surgery [1]. This publication provides a step-by-step approach that includes clinical evaluation with risk factors assessment, test findings, estimated outcomes and the burden of surgery, as well as the risks of discontinuing medications. The leading purpose of this approach is to optimize the perioperative patient’s state. Consequently, the guidelines aim to specifically assess risk with the initiation of drug therapy, cardiac manipulation, specific anesthetic, and surgical modalities when necessary, or avoidance of certain medications [1].

Numerous studies recognize the importance of functional capacity evaluation as a common element of preoperative assessment before extensive, especially non-cardiac, surgery. The aim of this assessment is to identify a high risk of critical postoperative complications. Preoperative stress testing is widely used to evaluate functional capacity in patients before non-cardiac surgeries. However, its value in predicting perioperative mortality is unclear. This paper will focus on the following questions: “Do we use preoperative stress testing rationally?”; “Do these methods have limited diagnostic and prognostic value in surgical patients?”; “Are we using these methods incorrectly?” and “Do we assess additional parameters that could improve the quality of life and prognosis in patients undergoing non-cardiac procedures?”.

## 2. Before Non-Cardiac Surgery: For Whom is Stress Testing Necessary and When?

A significant percentage of patient-related risks depends on cardiovascular diseases (CVD) and their risk factors (smoking, high blood pressure, diabetes, dyslipidemia, and family history), age, and comorbidities. In addition, it is crucial to identify the presence or absence of established cardiovascular conditions [2].

**Fig. 1.**
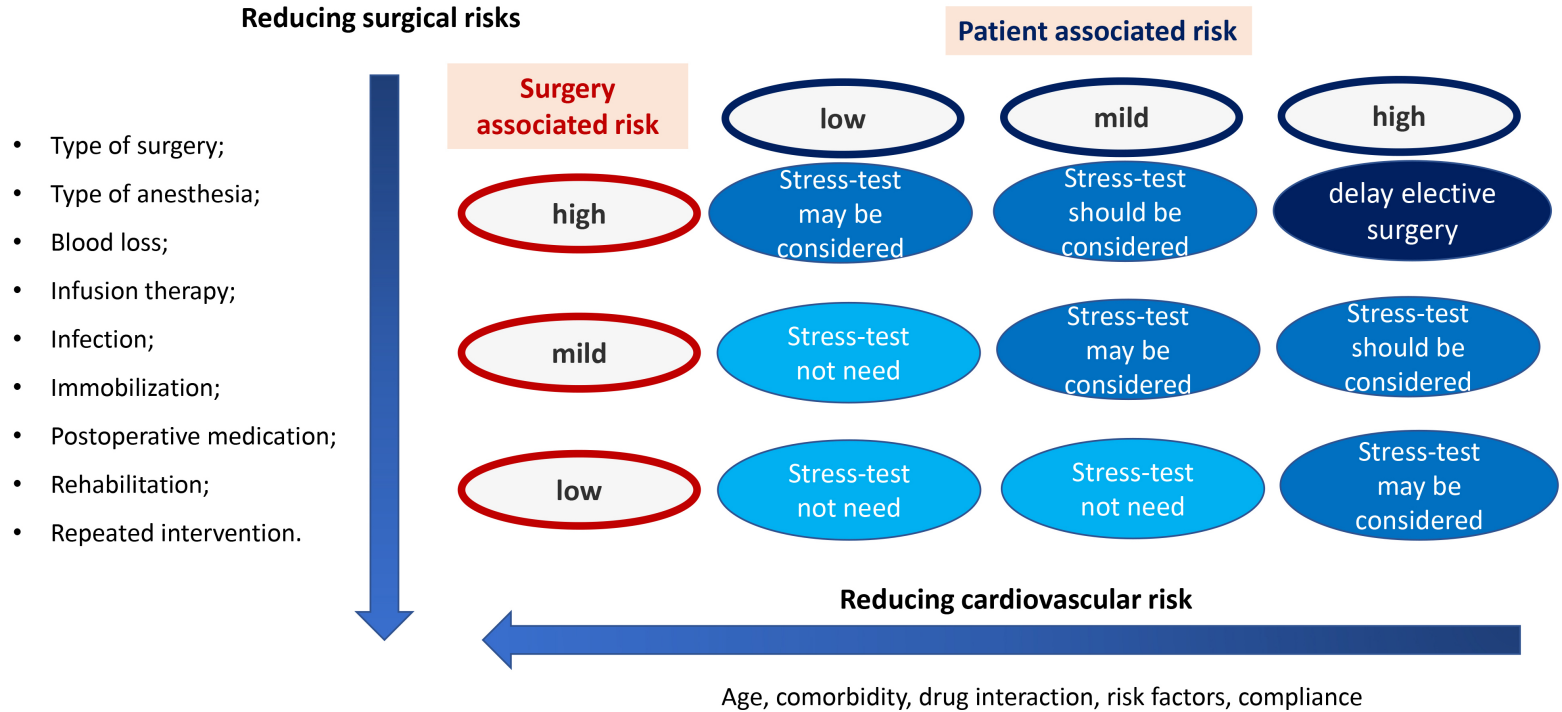
**The summary of factors that are important to take into account during non-cardiac surgery**. Adapted from 2022 ESC Guidelines on cardiovascular assessment and management of patients undergoing non-cardiac surgery [1].

Generally, not only are the surgery risks associated with the patient’s state or comorbidities, but also with the type, length of time, and importance of the operation, as well as the choice of anesthesia. Anesthesia methods and medication may affect intermediate to high cardiac risk patients undergoing non-cardiac surgery [3]. Neuraxial anesthesia, such as intrathecal or epidural anesthesia, is viewed as a high-risk intervention with a limited positive outcome in most patients. Furthermore, bleeding complications associated with potentially elevated direct oral anticoagulant levels, especially neuraxial hematoma, could be devastating. The evidence remains limited for the optimal perioperative or peri-interventional treatment despite the increasing number of patients chronically treated with anticoagulants and antiplatelet drugs, especially in urgent or emergent surgery [4]. Fig. 1 (Ref. [1]) shows the summary of factors that are important to take into account during non-cardiac surgery.

**Fig. 2.**
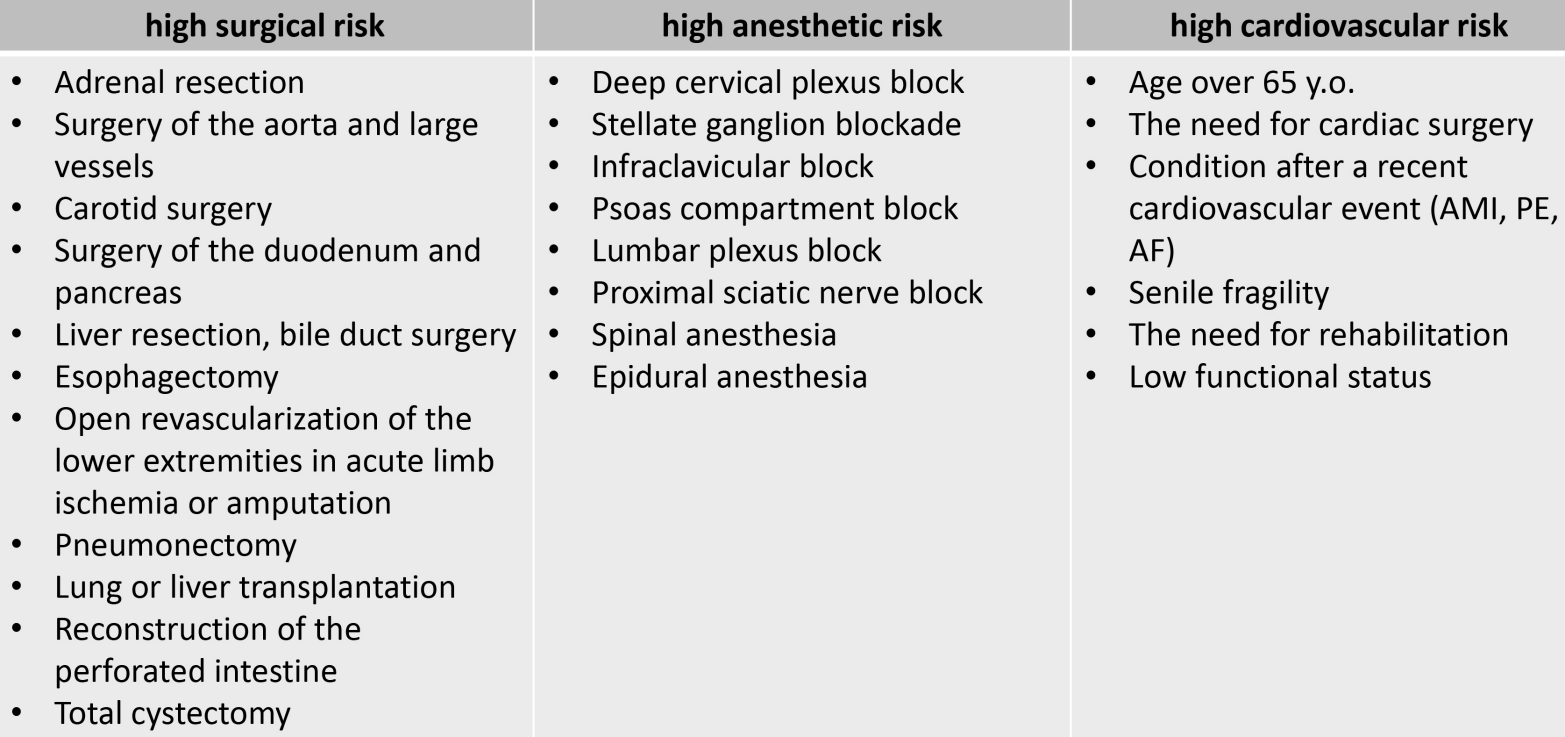
**High risk patients before non-cardiac surgery**. Adapted from 2022 ESC Guidelines on cardiovascular assessment and management of patients undergoing non-cardiac surgery [1]. AMI, acute myocardial infarction; PE, pulmonary embolism; AF, atrial fibrillation.

**Fig. 3.**
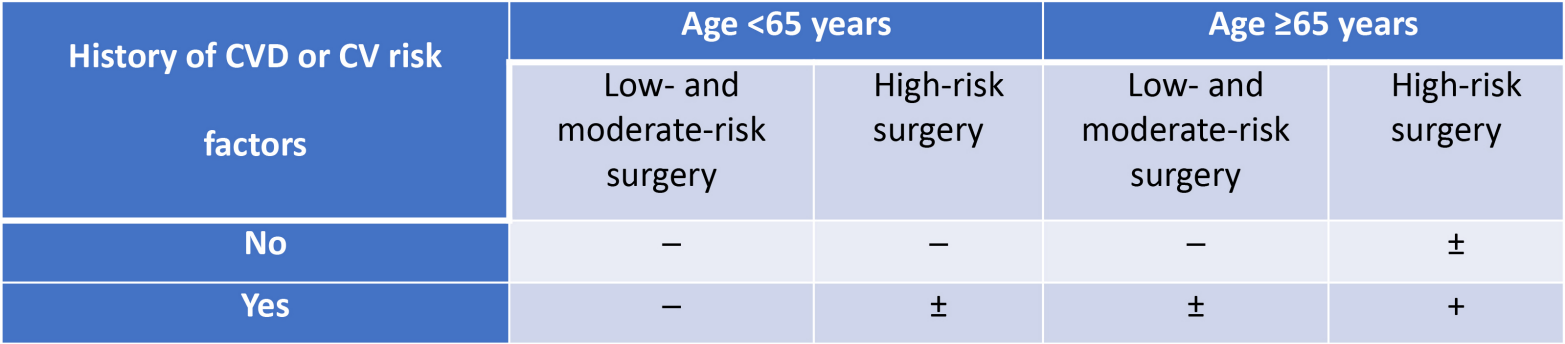
**Stress testing usage before non-cardiac surgery: ─ (not recommended), ± (should/may be considered), + (should/may be performed)**. Adapted from 2022 ESC Guidelines on cardiovascular assessment and management of patients undergoing non-cardiac surgery [1]. CVD, cardiovascular diseases; CV, cardiovascular.

Stress testing is a significant area of interest within the field of detailed patient functional capacity evaluation. Based on stress test findings, clinicians may further assess risks associated with the patient’s state and the surgery. Moreover, patients at low risk younger than 65 years without any complaints and known cases of cardiovascular events or risk factors may not require preoperative risk extra examination before low- and moderate-risk procedures [5]. While in the case of high-risk surgery, low-risk patients should only have an electrocardiogram (ECG) and biomarker examination. On the other hand, individuals need extra examination before intermediate and high-risk procedures, as well as risk factor management if they are older than 65 years or have cardiovascular risk factors. Patients with hypertension, dyslipidemia, or smoking have an increased risk of perioperative complications during non-cardiac surgery [6]. Fig. 2 (Ref. [1]) presents a total summary of high-risk patients, and Fig. 3 (Ref. [1]) describes stress testing usage before non-cardiac surgery.

In clinical practice, there are various types of stress testing. The use of methods and the choice of the optimal one depends on the study purpose and the availability of hospital equipment. Below we will discuss the main types of stress tests that we can use before non-cardiac surgery.

## 3. Exercise Electrocardiography

Traditionally, the value of exercise ECG, or ECG stress test, has subscribed to the belief that it is beneficial for perioperative cardiac risk prediction. However, in the last two decades, the key role of this method has lost its importance due to the growing trend toward cardiac imaging techniques [7]. Nevertheless, the published literature describing the predictive value of exercise electrocardiogram compared with clinical data and resting electrocardiography has highlighted that ST segment depression of 0.1 mV or more on the exercise electrocardiogram is an independent predictor of perioperative cardiac complications [7]. The authors published these data more than 20 years ago, and their work is the only one proving the feasibility of ECG testing before non-cardiac surgery.

Considering the latest diagnostic options and cardiac imaging, the niche of ECG stress tests is very narrow. On the one hand, the treadmill or bicycle ergometer exercises allow specialists to assess functional capability, blood pressure, and heart rate, as well as identify suspicion of myocardial ischemia by ST segment changes. On the other hand, the predictive power of the exercise ECG test varies significantly in different studies [8]. Moreover, exercise testing for risk stratification in patients with physical disabilities is inappropriate due to the inability to reach their target heart rate. In addition, pre-existing resting ST segment abnormalities, especially in precordial V5 and V6 leads, make reliable ST segment analysis difficult. Thus, the onset of an ischemic myocardial reaction with low physical activity correlates with a critically increased risk of perioperative and long-term cardiac events. In contrast, the myocardial ischemia occurring with high physical activity relates to a slightly higher risk compared with a normal test result [9]. Besides, the lack of myocardial and intracardiac structures imaging and the cardiac test’s objective control makes the ECG test less desirable for assessing structural changes. However, in the last two decades, the key role of this method has lost its importance due to the growing trend toward cardiac imaging techniques [7]. Considering the possible unavailability of non-invasive imaging tests in hospitals, nothing other than an ECG test can be useful for coronary heart disease diagnostics or can help to evaluate exercise tolerance when clinical history is questionable [10].

## 4. The Six-Minute-Walk Test

Usually, physicians collect patients’ medical histories during an interview to assess their functionality. Moreover, clinicians should be vigilant with patients who plan surgery because they can intentionally hide symptoms that may interfere with the operation and unintentionally underestimate their importance. Therefore, patients’ self-assessment of functional abilities is not a specific indicator of perioperative risk [11].

The six-minute walk test (6MWT) would be a more acceptable alternative for risk stratification. It is a simple-to-perform, well-tolerated, inexpensive, and clinically proven indicator of cardiopulmonary status. The objective of the 6MWT is to measure the distance walked for six minutes. The test requires a 30-meter stretch unimpeded track and standardized encouragement. Data from several studies suggest that the six-minute walk test is a safe and robust indicator of physical function and correlates with cardiopulmonary exercise testing (CPET) findings. In addition, the 6MWT has significantly rare serious side effects [12]. Despite existing research, there is still no evidence about the link between 6MWT distance and complications. A considerable amount of literature has been published on the topic, but the findings are controversial [12]. Thus, further large high-quality studies are vital to generating new insight into the role of 6MWT in pre-operative risk stratification.

Some authors investigated the 6MWT in patients with lung cancer before a major elective non-cardiac surgery. Nevertheless, in 2015 Marjanski *et al*. [13] published a research paper in which they examined 253 lung cancer patients who indicated lobectomy. In addition to the routine protocol, they performed the 6MWT one day before the procedure. According to the 6MWT findings, the research team grouped patients to evaluate postoperative complication risk. The result of the study demonstrated that patients have higher postoperative risk and stay longer in a hospital if they walk less than 500 m during the six-minute walk test before the surgery [13].

Upon further analysis of this multicenter prospective cohort study, researchers noted a decrease in the preoperative 6MWT distance in some patients. This decrease was associated with an increase in moderate or severe in-hospital complications, even after adjusting to other easily measurable clinical characteristics, such as demographic data and type of surgery. However, the statistical reliability of this association was limited. Investigators have found no proof that 6MWT provides additional prognostic information for predicting postoperative complications. As a comparison, 6MWT distance had no significant association with myocardial injury or 30-day postoperative death. These overall results were consistent after controlling for other preoperative risk factors and internal validation using bootstrap resampling.

Moreover, Sinclair *et al*. [14] hold the view that the six min walk test is a reliable and effective substitute for perioperative risk stratification in case of unavailable CPET. The researchers assumed the benefit of 6MWT distance assessment for low and high anaerobic threshold determination before the extended non-cardiac procedure. In 2012 they found that people with a 6MWT distance of more than 563 m do not usually need cardiopulmonary exercise testing. On the other side, individuals with a 6MWT distance of fewer than 427 m require additional examination. Herewith, in unclear cases when patients walk more than 427 m but less than 563 m, physicians should consider some risk factors and the extent of surgical procedures [14]. Overall, the 6MWT can help to identify risk factors for the patients before the surgical intervention if CPET is unavailable.

According to current trends, the 6MWT is a helpful tool in preoperative assessment and plays a critical role in post-surgical rehabilitation [15]. Recent findings have shown how 6MWT can influence the postoperative complication risk [16]. These results indicate the need to understand the usefulness of the six-minute walk test in surgical patients before the non-cardiac procedure, especially at the screening stage.

## 5. Cardiopulmonary Exercise Testing

Historically, clinicians have been detecting poor functional capacity for years by subjective assessment if patients have less than four metabolic equivalents. However, as we have mentioned before, these interviews are an unreliable and imprecise option to reveal poor exercise tolerance and predict postoperative outcomes. In many clinics, the functional capacity examination has become a key aspect of preoperative cardiac risk stratification [9]. Despite patients’ interviews about their fitness shortcomings, some authors have recently reported that high-risk individuals undergoing non-cardiac surgery have additional utility for risk stratification if they announce less than two flight stairs climbing inability [17]. In 2018, Wijeysundera *et al*. [18] demonstrated the value of the Duke Activity Status Index (DASI) by showing patient-reported physical activity. This DASI questionnaire produces more accurate information about cardiac risk and improves examination for surgical patients [18]. Besides, the result correlated with maximal oxygen consumption (${\mathrm{VO}}_{2}$max) and metabolic equivalents: ${\mathrm{VO}}_{2}$ max (mL/kg/min) = 0.43 × DASI + 9.6 and metabolic equivalents = ${\mathrm{VO}}_{2}$max/3.5. Moreover, a DASI score of less than 34 is linked to increased 30-day mortality or myocardial infarction. However, the CPET did not predict that. Notably, some studies had few primary outcomes, which limited the analysis [18].

When combined with exercise testing, adjunctive imaging modalities offer greater diagnostic accuracy, additional information regarding cardiac structure and function, and additional prognostic information. Similarly, additional measurements of ventilatory gas exchange during exercise testing provide a wide array of unique and clinically useful incremental information that has been poorly understood and underutilized by practitioners [18]. Cardiopulmonary exercise testing, as a dynamic clinical tool, detects patients’ cardiorespiratory state. It has a lot of clinical indications that include therapy evaluation, stratification of risk factors, disease diagnostics, and physical activity control. Traditionally, we use treadmills or bicycle ergometers to estimate exercise tolerance, but cardiopulmonary exercise testing, as a type of dedicated exercise testing, can demonstrate particular and detailed cardiorespiratory fitness at rest and under stress.

The CPET is a non-invasive method that measures ventilatory gases, heart rate, and blood pressure during exercise. As a golden standard for exercise performance assessment, it provides information about exhaled air and the oxygen ($O_{2}$) and carbon dioxide (${\mathrm{CO}}_{2}$) concentration during increased activity. However, patients may have some obstacles related to the testing itself—anxiety, equipment issues, etc.—that limit this approach. In addition, this test may demonstrate accurate and effective cardiorespiratory capacity, etiology, severity classification, and treatment response [19].

During the past 20 years, much more information has become available on cardiopulmonary exercise testing as a prognostic method. CPET helps the clinician to obtain a multitude of information beyond standard exercise electrocardiography testing. Thus, appropriately applied and interpreted CPET can assist in complex cardiovascular and pulmonary disease management [19]. It has conclusively been shown that cardiopulmonary exercise testing results are essential for further risk stratification before being placed on a waiting list for a heart transplant or medical device insertion, like cardioverter-defibrillator and cardiac resynchronization therapy. Besides, as mentioned in the literature, this approach plays a vital role in providing additional information for lung resection or transplantation and other preoperative conditions [20, 21, 22, 23, 24].

It is now well established from a variety of studies that reduced exercise tolerance leads to increased negative postoperative outcomes. In addition, cardiopulmonary exercise testing can help to evaluate preoperative risks and predict postoperative outcomes [25, 26]. Prior studies noted the importance of CPET parameters in risk prediction [18, 25]. The systematic review performed by Moran *et al*. [25] demonstrated the influence of CPET results on prognosis and prediction of postoperative outcomes after major non-cardiac surgery.

Taking into account the increase in life expectancy of the population and the increase in patients with comorbidity, the CPET before elective surgery will allow identifying new cases of heart failure and preparing the patient for non-cardiac surgery, minimizing the risks of cardiovascular complications.

## 6. Stress Imaging Tests

For patients with clinical risk factors and weak functional capacity, it is optimal to perform stress imaging [27]. According to clinical potentials, the type of chosen method should be considered. Additionally, as provided by current guidelines and recommendations, physicians should not use stress imaging for patients in need of urgent surgery or unstable state [1, 28]. Stress imaging is one of the most practical ways of perioperative risk prediction and patient management. The test is based on ischemia provoked by pharmacological agents or exercise. In the literature, there are no established conformations about the significant advantages of pharmacological stress compared with exercise stress imaging in people who are capable enough to be physically active.

Despite the lack of randomized controlled trials aimed at surgery outcomes, other large prospective studies demonstrated the link between stress testing findings and perioperative cardiac complications [29, 30]. Thus, meta-analyses of Beattie *et al*. [29] identified that pharmacological stress imaging has predictive characteristics for perioperative risk evaluation in individuals undergoing non-cardiac surgery. Moreover, some researchers revealed the dependence of stress echocardiography on clinical predictive usefulness for risk assessment and ischaemic heart disease prevalence [29, 31]. The results of the stress imaging test are useful in the diagnosis of latent cardiovascular diseases that do not manifest themselves at rest, and symptoms appear only when performing physical activities. The results obtained during stress imaging may indicate the absence or presence of deviations. While the risk of perioperative complications is lower among people without identified pathology, patients with identified changes using stress imaging need to re-stratify the cardiac risk of extra-cardiac surgery. When performing a visualizing stress test, pulmonary hypertension may be detected, which was not detected during the study at rest, or increased valvular regurgitation and/or the appearance of B-lines [31, 32].

The results of previous studies regarding the role of dobutamine stress echocardiography in risk assessment in non-cardiac surgical patients have shown additional prognostic usage [29]. Moreover, the risk of perioperative cardiac events can be stratified after dobutamine stress echocardiography, which allows to determination the presence of myocardial ischemia and heart rate at ischemia (ischemic threshold) [33, 34].

Patients undergoing dobutamine stress echocardiography before major non-cardiac surgery may not achieve their target heart rate even with a high-dose protocol. Patients performing negative test results and lack of wall motion abnormality at rest demonstrated significant negative predictive value [35]. In asymptomatic patients, who are aware of their physical tolerance, exercise echocardiography can assess myocardial function (systolic and diastolic), valvular pathologies, and high pulmonary artery pressure [36]. In these cases, exercise-imaging testing would be one of the most informative techniques for diagnostics. However, insufficient information is available about the dobutamine stress test’s role in non-cardiac preoperative risk evaluation. This would be a fruitful area for further research.

Myocardial perfusion imaging may be the method of choice in the setting of suboptimal echocardiography imaging. In addition, in some studies, when major non-cardiac surgery was performed, a higher risk of cardiac events was noted in persons with reversible perfusion defects compared to fixed defects [30, 37, 38]. Besides, stress cardiac magnetic resonance imaging (MRI) and contrast MRI are reliable options for coronary artery disease and prognosis detection [39].

## 7. Stress Testing before Non-Cardiac Surgery: What is the Optimal Protocol?

Current guidelines of various communities (European Society of Cardiology [ESC], American College of Cardiology [ACC], Canadian College of Cardiology [CCS]) provide summary data results of various preoperative exercise tests in patients before non-cardiac surgical interventions. However, experts have no consensus on the appropriateness of these methods in identifying risk groups for potential perioperative complications. A large volume of published studies describes the role of preoperative exercise stress testing in improving the prediction of potential CV risk in patients after non-cardiac surgery. Nevertheless, the overall number of observations and events was low [37, 40, 41, 42, 43]. When physicians/sonographers perform stress echocardiography to identify predictors of perioperative complications, not only is the study concerned, but also the conditions and methodology (choice of stress agent and stress-testing protocol) are important [26, 37, 43, 44, 45, 46, 47].

A large prospective cohort study including 1725 patients undergoing planned major abdominal or thoracic surgery revealed that modern exercise tests are weak independent predictors of perioperative cardiac complications [44]. Other studies have shown similar results, and none have determined whether the effectiveness of exercise testing improves risk reclassification in addition to clinical evaluation [26, 37, 40, 41, 42, 43, 44, 45, 46, 47].

Exercise stress echocardiography is the method of choice for most stress test protocols. This method preserves the integrity of the picture of the relationship between hemodynamic changes and clinical symptoms. Moreover, it provides valuable information about the functional patient state [47]. Echocardiography during exercise can establish associations between symptoms, cardiovascular stress, wall motion abnormalities, and hemodynamic responses, such as pulmonary artery pressure and transvalvular flows and gradients [48, 49]. Echocardiography can be performed on a treadmill, bicycle ergometer, upper body ergometer, or step platform [36]. The choice of an ergometer type depends on each medical institution’s facilities. On the other hand, some constitutional patients’ features also influence the choice of ergometric methods. Thus, the upper body diagnostic stress systems seem relevant for patients with knee and hip disorders.

The potential role of telemonitoring is an important tool for real-time heart monitoring. Overall, the incidence of postoperative atrial fibrillation (AF) during hospitalization ranged from 3% to 30%. AF incidence varied with the type of surgery [50]. Prospective studies using continuous ECG monitoring reported significantly higher incidences of AF than those that did not (13.9% vs 1.9%, respectively; *p *< 0.001) [51]. Given the widespread implementation of mobile telemonitoring, it is important to take care of patients both before and after non-cardiac surgery.

Cardiac troponins (cTns) are the most valuable and specific markers of cardiovascular diseases, including acute myocardial infarction. Natriuretic peptides (B-type natriuretic peptide [BNP] and N-terminal pro B-type natriuretic peptide [NT-proBNP]) are crucial in heart failure diagnostics. These biomarkers can also assess the degree of myocardial damage in non-cardiac diseases that can negatively affect the cells of cardiac muscle tissue. However, in everyday clinical practice, doctors often encounter false-positive cases of increased levels of these biomarkers. False-positive cases of increased cTns or natriuretic peptides can contribute to incorrect diagnosis and subsequent inadequate treatment, which causes significant harm to the patient. Physicians and researchers should also keep in mind a considerable number of factors provoking false-positive elevations in biomarkers of cardiac injury, as well as ways to detect false-positive results and counteract them [52]. Additionally, combining NT-proBNP with a specific risk score (e.g., Geriatric-Sensitive Cardiac Risk Index) can improve discriminatory ability in elderly patients before vascular surgery [53]. Therefore, stress tests look to be an important addition when assessing risks in elective surgical patients.

Based on the current recommendations, it is possible to use imaging stress tests in patients who are planning a high-risk intervention or with a reduced or unknown status of functional activity. There are increased requirements for stress echocardiography: now it is not only the diagnosis of ischemic disorders and areas of impaired myocardial contractility, but also the function of valves, regurgitation, chamber sizes in response to load, and, of course, patient’s symptoms. Routine stress testing for each patient before non-cardiac surgery is a genuine burden on the healthcare system. Recently, the largest meta-analysis on this problem has been conducted, leaving more questions than answers about the feasibility of stress imaging before non-cardiac surgeries. Firstly, researchers have conducted 36 out of 40 studies without a comparison group. This situation indicates low methodological quality. Secondly, according to this meta-analysis, the risk of 30-day postoperative mortality associated with positive stress test results compared with negative preoperative test results did not give statistically significant differences. Third, of the 1807 studies reviewed for this analysis, 485 (26.8%) were excluded because they did not assess outcomes such as mortality, myocardial infarction rate, or heart failure rate. Therefore, this meta-analysis concluded that, despite the considerable interest and research conducted over the past 40 years to predict the 30-day mortality risk among patients undergoing non-cardiac surgery, the available data are insufficient to make a definitive conclusion about whether stress testing leads to an improvement in the assessment of perioperative risk [54]. All of these studies examined a stress test for induced myocardial ischemia. Currently, we lack data about ischemic stress tests use, let alone diastolic in perioperative risk assessment, before non-cardiac surgical interventions. We consider it promising and essential to continue research in this field with patients with coronary heart disease and other categories of cardiac patients, in particular, comorbid and low-symptomatic individuals, before high-risk elective surgical interventions [55].

## 8. Conclusions 

The prognosis of the patients in the perioperative phase of non-cardiac surgery strongly depends on cardiovascular outcomes. Now it is necessary to thoroughly select a suitable technique of cardiac stress testing for non-cardiac risk stratification following the patient’s state, type of operation, desirable information, hospital resources, and diagnostic effectiveness. Thus, clinicians should use a versatile strategy for assessing people before planned non-cardiac surgery and decide whether the management of cardiac pathologies will improve perioperative prognosis.

## References

[1] Halvorsen S, Mehilli J, Cassese S, Hall TS, Abdelhamid M, Barbato E (2022). 2022 ESC Guidelines on cardiovascular assessment and management of patients undergoing non-cardiac surgery. *European Heart Journal*.

[2] Visseren FLJ, Mach F, Smulders YM, Carballo D, Koskinas KC, Bäck M (2021). 2021 ESC Guidelines on cardiovascular disease prevention in clinical practice. *European Heart Journal*.

[3] Bolliger M, Kroehnert JA, Molineus F, Kandioler D, Schindl M, Riss P (2018). Experiences with the standardized classification of surgical complications (Clavien-Dindo) in general surgery patients. *European Surgery*.

[4] Kietaibl S, Ferrandis R, Godier A, Llau J, Lobo C, Macfarlane AJ (2022). Regional anaesthesia in patients on antithrombotic drugs: Joint ESAIC/ESRA guidelines. *European Journal of Anaesthesiology*.

[5] Botto F, Alonso-Coello P, Chan MTV, Villar JC, Xavier D, Srinathan S (2014). Myocardial injury after noncardiac surgery: a large, international, prospective cohort study establishing diagnostic criteria, characteristics, predictors, and 30-day outcomes. *Anesthesiology*.

[6] Chaudhry W, Cohen MC (2017). Cardiac Screening in the Noncardiac Surgery Patient. *The Surgical Clinics of North America*.

[7] Priebe HJ (2011). Preoperative cardiac management of the patient for non-cardiac surgery: an individualized and evidence-based approach. *British Journal of Anaesthesia*.

[8] Montalescot G, Sechtem U, Achenbach S, Andreotti F, Arden C, Task Force Members (2013). 2013 ESC guidelines on the management of stable coronary artery disease: the Task Force on the management of stable coronary artery disease of the European Society of Cardiology. *European Heart Journal*.

[9] Kristensen SD, Knuuti J, Saraste A, Anker S, Bøtker HE, De Hert S (2014). 2014 ESC/ESA Guidelines on non-cardiac surgery: cardiovascular assessment and management: The Joint Task Force on non-cardiac surgery: cardiovascular assessment and management of the European Society of Cardiology (ESC) and the European Society of Anaesthesiology (ESA). *European Journal of Anaesthesiology*.

[10] Knuuti J, Wijns W, Saraste A, Capodanno D, Barbato E, Funck-Brentano C (2020). 2019 ESC Guidelines for the diagnosis and management of chronic coronary syndromes. *European Heart Journal*.

[11] Ramos RJ, Ladha KS, Cuthbertson BH, Shulman MA, Myles PS, Wijeysundera DN (2021). Association of six-minute walk test distance with postoperative complications in non-cardiac surgery: a secondary analysis of a multicentre prospective cohort study. *Canadian Journal of Anaesthesia*.

[12] Singh SJ, Puhan MA, Andrianopoulos V, Hernandes NA, Mitchell KE, Hill CJ (2014). An official systematic review of the European Respiratory Society/American Thoracic Society: measurement properties of field walking tests in chronic respiratory disease. *The European Respiratory Journal*.

[13] Marjanski T, Wnuk D, Bosakowski D, Szmuda T, Sawicka W, Rzyman W (2015). Patients who do not reach a distance of 500 m during the 6-min walk test have an increased risk of postoperative complications and prolonged hospital stay after lobectomy. *European Journal of Cardio-thoracic Surgery*.

[14] Sinclair RCF, Batterham AM, Davies S, Cawthorn L, Danjoux GR (2012). Validity of the 6 min walk test in prediction of the anaerobic threshold before major non-cardiac surgery. *British Journal of Anaesthesia*.

[15] Machała E, Redynk M, Gruchała A, Kołomecki K (2020). Analysis of exercise tolerance on the basis of six-minute walk test - 6MWT and Borg RPE scale in men with inguinal hernia before and after Lichtenstein repair. *Polski Przeglad Chirurgiczny*.

[16] Marjanski T, Wnuk D, Dziedzic R, Ostrowski M, Sawicka W, Rzyman W (2021). 500 Meters Is a Result of 6-Minute Walk Test Which Differentiates Patients with High and Low Risk of Postoperative Complications after Lobectomy-A Validation Study. *Journal of Clinical Medicine*.

[17] Lurati Buse GAL, Puelacher C, Gualandro DM, Genini AS, Hidvegi R, Bolliger D (2021). Association between self-reported functional capacity and major adverse cardiac events in patients at elevated risk undergoing noncardiac surgery: a prospective diagnostic cohort study. *British Journal of Anaesthesia*.

[18] Wijeysundera DN, Pearse RM, Shulman MA, Abbott TEF, Torres E, Ambosta A (2018). Assessment of functional capacity before major non-cardiac surgery: an international, prospective cohort study. *Lancet*.

[19] Guazzi M, Adams V, Conraads V, Halle M, Mezzani A, Vanhees L (2012). EACPR/AHA Scientific Statement. Clinical recommendations for cardiopulmonary exercise testing data assessment in specific patient populations. *Circulation*.

[20] Mezzani A, Agostoni P, Cohen-Solal A, Corrà U, Jegier A, Kouidi E (2009). Standards for the use of cardiopulmonary exercise testing for the functional evaluation of cardiac patients: a report from the Exercise Physiology Section of the European Association for Cardiovascular Prevention and Rehabilitation. *European Journal of Cardiovascular Prevention and Rehabilitation*.

[21] Palange P, Ward SA, Carlsen KH, Casaburi R, Gallagher CG, Gosselink R (2007). Recommendations on the use of exercise testing in clinical practice. *The European Respiratory Journal*.

[22] Arena R, Myers J, Guazzi M (2008). The clinical and research applications of aerobic capacity and ventilatory efficiency in heart failure: an evidence-based review. *Heart Failure Reviews*.

[23] Arena R, Myers J, Williams MA, Gulati M, Kligfield P, Balady GJ (2007). Assessment of functional capacity in clinical and research settings: a scientific statement from the American Heart Association Committee on Exercise, Rehabilitation, and Prevention of the Council on Clinical Cardiology and the Council on Cardiovascular Nursing. *Circulation*.

[24] Brunelli A, Belardinelli R, Refai M, Salati M, Socci L, Pompili C (2009). Peak oxygen consumption during cardiopulmonary exercise test improves risk stratification in candidates to major lung resection. *Chest*.

[25] Moran J, Wilson F, Guinan E, McCormick P, Hussey J, Moriarty J (2016). Role of cardiopulmonary exercise testing as a risk-assessment method in patients undergoing intra-abdominal surgery: a systematic review. *British Journal of Anaesthesia*.

[26] Lai CW, Minto G, Challand CP, Hosie KB, Sneyd JR, Creanor S (2013). Patients’ inability to perform a preoperative cardiopulmonary exercise test or demonstrate an anaerobic threshold is associated with inferior outcomes after major colorectal surgery. *British Journal of Anaesthesia*.

[27] Pellikka PA, Arruda-Olson A, Chaudhry FA, Chen MH, Marshall JE, Porter TR (2020). Guidelines for Performance, Interpretation, and Application of Stress Echocardiography in Ischemic Heart Disease: From the American Society of Echocardiography. *Journal of the American Society of Echocardiography*.

[28] Wolk MJ, Bailey SR, Doherty JU, Douglas PS, Hendel RC, Kramer CM (2014). ACCF/AHA/ASE/ASNC/HFSA/HRS/SCAI/SCCT/SCMR/STS 2013 multimodality appropriate use criteria for the detection and risk assessment of stable ischemic heart disease: a report of the American College of Cardiology Foundation Appropriate Use Criteria Task Force, American Heart Association, American Society of Echocardiography, American Society of Nuclear Cardiology, Heart Failure Society of America, Heart Rhythm Society, Society for Cardiovascular Angiography and Interventions, Society of Cardiovascular Computed Tomography, Society for Cardiovascular Magnetic Resonance, and Society of Thoracic Surgeons. *Journal of the American College of Cardiology*.

[29] Beattie WS, Abdelnaem E, Wijeysundera DN, Buckley DN (2006). A meta-analytic comparison of preoperative stress echocardiography and nuclear scintigraphy imaging. *Anesthesia and Analgesia*.

[30] Cullen MW, McCully RB, Widmer RJ, Schroeder DR, Salonen BR, Raslau D (2020). Preoperative Dobutamine Stress Echocardiography and Clinical Factors for Assessment of Cardiac Risk after Noncardiac Surgery. *Journal of the American Society of Echocardiography*.

[31] Cohen MC, Siewers AE, Dickens JD, Hill T, Muller JE (2003). Perioperative and long-term prognostic value of dipyridamole Tc-99m sestamibi myocardial tomography in patients evaluated for elective vascular surgery. *Journal of Nuclear Cardiology*.

[32] Dowsley TF, Sheth T, Chow BJW (2020). Complementary pre-operative risk assessment using coronary computed tomography angiography and nuclear myocardial perfusion imaging in non-cardiac surgery: A VISION-CTA sub-study. *Journal of Nuclear Cardiology*.

[33] Torres MR, Short L, Baglin T, Case C, Gibbs H, Marwick TH (2002). Usefulness of clinical risk markers and ischemic threshold to stratify risk in patients undergoing major noncardiac surgery. *The American Journal of Cardiology*.

[34] Das MK, Pellikka PA, Mahoney DW, Roger VL, Oh JK, McCully RB (2000). Assessment of cardiac risk before nonvascular surgery: dobutamine stress echocardiography in 530 patients. *Journal of the American College of Cardiology*.

[35] Labib SB, Goldstein M, Kinnunen PM, Schick EC (2004). Cardiac events in patients with negative maximal versus negative submaximal dobutamine echocardiograms undergoing noncardiac surgery: importance of resting wall motion abnormalities. *Journal of the American College of Cardiology*.

[36] Lancellotti P, Pellikka PA, Budts W, Chaudhry FA, Donal E, Dulgheru R (2016). The clinical use of stress echocardiography in non-ischaemic heart disease: recommendations from the European Association of Cardiovascular Imaging and the American Society of Echocardiography–European Heart Journal. *Cardiovascular Imaging*.

[37] Etchells E, Meade M, Tomlinson G, Cook D (2002). Semiquantitative dipyridamole myocardial stress perfusion imaging for cardiac risk assessment before noncardiac vascular surgery: a meta-analysis. *Journal of Vascular Surgery*.

[38] Metz LD, Beattie M, Hom R, Redberg RF, Grady D, Fleischmann KE (2007). The prognostic value of normal exercise myocardial perfusion imaging and exercise echocardiography: a meta-analysis. *Journal of the American College of Cardiology*.

[39] Nandalur KR, Dwamena BA, Choudhri AF, Nandalur MR, Carlos RC (2007). Diagnostic performance of stress cardiac magnetic resonance imaging in the detection of coronary artery disease: a meta-analysis. *Journal of the American College of Cardiology*.

[40] Kaaja R, Sell H, Erkola O, Harjula A (1993). Predictive value of manual ECG-monitored exercise test before abdominal aortic or peripheral vascular surgery. *Angiology*.

[41] McPhail N, Calvin JE, Shariatmadar A, Barber GG, Scobie TK (1988). The use of preoperative exercise testing to predict cardiac complications after arterial reconstruction. *Journal of Vascular Surgery*.

[42] Carliner NH, Fisher ML, Plotnick GD, Garbart H, Rapoport A, Kelemen MH (1985). Routine preoperative exercise testing in patients undergoing major noncardiac surgery. *The American Journal of Cardiology*.

[43] Sgura FA, Kopecky SL, Grill JP, Gibbons RJ (2000). Supine exercise capacity identifies patients at low risk for perioperative cardiovascular events and predicts long-term survival. *The American Journal of Medicine*.

[44] Colson M, Baglin J, Bolsin S, Grocott MPW (2012). Cardiopulmonary exercise testing predicts 5 yr survival after major surgery. *British Journal of Anaesthesia*.

[45] Grant SW, Hickey GL, Wisely NA, Carlson ED, Hartley RA, Pichel AC (2015). Cardiopulmonary exercise testing and survival after elective abdominal aortic aneurysm repair†. *British Journal of Anaesthesia*.

[46] Hartley RA, Pichel AC, Grant SW, Hickey GL, Lancaster PS, Wisely NA (2012). Preoperative cardiopulmonary exercise testing and risk of early mortality following abdominal aortic aneurysm repair. *The British Journal of Surgery*.

[47] Duceppe E, Parlow J, MacDonald P, Lyons K, McMullen M, Srinathan S (2017). Canadian Cardiovascular Society Guidelines on Perioperative Cardiac Risk Assessment and Management for Patients Who Undergo Noncardiac Surgery. *The Canadian Journal of Cardiology*.

[48] Wijeysundera DN, Beattie WS, Karkouti K, Neuman MD, Austin PC, Laupacis A (2011). Association of echocardiography before major elective non-cardiac surgery with postoperative survival and length of hospital stay: population based cohort study. *British Medical Journal*.

[49] Cortigiani L, Rigo F, Gherardi S, Galderisi M, Sicari R, Picano E (2008). Prognostic implications of coronary flow reserve on left anterior descending coronary artery in hypertrophic cardiomyopathy. *The American Journal of Cardiology*.

[50] McIntyre WF, Vadakken ME, Rai AS, Thach T, Syed W, Um KJ (2021). Incidence and recurrence of new-onset atrial fibrillation detected during hospitalization for non-cardiac surgery: a systematic review and meta-analysis. *Canadian Journal of Anaesthesia*.

[51] Bessissow A, Khan J, Devereaux PJ, Alvarez-Garcia J, Alonso-Coello P (2015). Postoperative atrial fibrillation in non-cardiac and cardiac surgery: an overview. *Journal of Thrombosis and Haemostasis*.

[52] Chaulin AM (2022). False-Positive Causes in Serum Cardiac Troponin Levels. *Journal of Clinical Medicine Research*.

[53] Perić VS, Golubović MD, Lazarević MV, Kostić TL, Stokanović DS, Đorđević MN (2021). Predictive potential of biomarkers and risk scores for major adverse cardiac events in elderly patients undergoing major elective vascular surgery. *Reviews in Cardiovascular Medicine*.

[54] Kalesan B, Nicewarner H, Intwala S, Leung C, Balady GJ (2019). Pre-operative stress testing in the evaluation of patients undergoing non-cardiac surgery: A systematic review and meta-analysis. *PLoS ONE*.

[55] Aitken R, Harun NS, Maier AB (2021). Which preoperative screening tool should be applied to older patients undergoing elective surgery to predict short-term postoperative outcomes? Lessons from systematic reviews, meta-analyses and guidelines. *Internal and Emergency Medicine*.

